# Identification of cryptolepine metabolites in rat and human hepatocytes and metabolism and pharmacokinetics of cryptolepine in Sprague Dawley rats

**DOI:** 10.1186/s40360-017-0188-8

**Published:** 2017-12-22

**Authors:** Arnold Donkor Forkuo, Charles Ansah, David Pearson, Werner Gertsch, Amanda Cirello, Adam Amaral, Jaimie Spear, Colin W. Wright, Caroline Rynn

**Affiliations:** 1 Department of Pharmacology, Faculty of Pharmacy and Pharmaceutical Science, College of Health Sciences Kwame Nkrumah University of Science and Technology, Kumasi, Ghana; 2 Drug Metabolism and Pharmacokinetics, Novartis Institutes for BioMedical Research, Novartis Pharma AG, Postfach, CH-4002 Basel, Switzerland; 3 Analytical Sciences and Imaging, Novartis Institutes for BioMedical Research, Novartis Pharma AG, Postfach, CH-4002 Basel, Switzerland; 4 Analytical Sciences and Imaging, Novartis Institutes for BioMedical Research, 250 Massachusetts Ave Cambridge, 02139 Cambridge, MA USA; 5 Metabolism and Pharmacokinetics, Novartis Institutes for BioMedical Research, 250 Massachusetts Ave Cambridge, 02139 Cambridge, MA USA; 6 School of Pharmacy, University of Bradford, West Yorkshire, BD7 1DP, Bradford, USA; 7 Metabolism and Pharmacokinetics, Novartis Institute for BioMedical Research, Novartis Pharma AG, Postfach, CH-4002 Basel, Switzerland

**Keywords:** Cryptolepine, *Cryptolepis sanguinolenta*, Metabolism, Pharmacokinetics, Metabolite identification

## Abstract

**Background:**

This study aims at characterizing the in vitro metabolism of cryptolepine using human and rat hepatocytes, identifying metabolites in rat plasma and urine after a single cryptolepine dose, and evaluating the single-dose oral and intravenous pharmacokinetics of cryptolepine in male Sprague Dawley (SD) rats.

**Methods:**

The in vitro metabolic profiles of cryptolepine were determined by LC-MS/MS following incubation with rat and human hepatocytes**.** The in vivo metabolic profile of cryptolepine was determined in plasma and urine samples from Sprague Dawley rats following single-dose oral administration of cryptolepine. Pharmacokinetic parameters of cryptolepine were determined in plasma and urine from Sprague Dawley rats after single-dose intravenous and oral administration.

**Results:**

Nine metabolites were identified in human and rat hepatocytes, resulting from metabolic pathways involving oxidation (M2-M9) and glucuronidation (M1, M2, M4, M8, M9). All human metabolites were found in rat hepatocyte incubations except glucuronide M1. Several metabolites (M2, M6, M9) were also identified in the urine and plasma of rats following oral administration of cryptolepine. Unchanged cryptolepine detected in urine was negligible. The Pharmacokinetic profile of cryptolepine showed a very high plasma clearance and volume of distribution (Vss) resulting in a moderate average plasma half-life of 4.5 h. Oral absorption was fast and plasma exposure and oral bioavailability were low.

**Conclusions:**

Cryptolepine metabolism is similar in rat and human in vitro with the exception of direct glucuronidation in human. Clearance in rat and human is likely to include a significant metabolic contribution, with proposed primary human metabolism pathways hydroxylation, dihydrodiol formation and glucuronidation. Cryptolepine showed extensive distribution with a moderate half-life.

## Background

Malaria is a major cause of morbidity and mortality, especially in Africa [1, 2]. The disease is commonly found in the tropical and subtropical regions of the world with about 214 million new cases and 438,000 deaths reported in 2015 worldwide [3]. According to the WHO [3], 90% of all malaria deaths reported in 2015 occurred in sub-Saharan Africa. Despite the reduction in malaria morbidity and mortality between 2000 and 2015, the major problem associated with further reduction or complete eradication of this disease is the increasing resistance of *Plasmodium falciparum* to most of the commonly used antimalarial drugs.

**Fig. 1:**
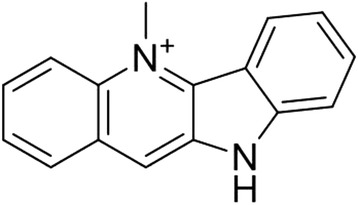
Structure of cryptolepine

Traditional medicines have been a major starting point for the development of antimalarial agents [4]. Quinine and artemisinin and their derivatives are important examples thereof [5]. *Cryptolepis sanguinolenta* (Lindl.) Schlechter is a popular Central- and West-African climbing shrub used over decades by African traditional healers for the treatment of fevers including malaria, hepatitis, bacterial infections and as antirheumatic and spasmolytic agents [6–8]. In Ghana, a clinical trial using a herbal tea bag preparation (containing *Cryptolepis sanguinolenta*) studied in forty four patients with clinical features of uncomplicated malaria showed more than half of the patients cleared of *P. falciparum* parasitaemia within 72 h (mean clearance = 82.3 h) [9]. The safety and efficacy studies of this popular antimalarial plant has brought hope to several millions of people who are affected by malaria in Ghana and other West African countries. On the Ghanaian market in 2015, there were fifteen (15) formulated herbal products containing *C. sanguinolenta* for the treatment of malaria (unpublished data). Cryptolepine (Fig. 1) is the major indoloquinoline alkaloid isolated from the plant and reported to possesses a number of pharmacological activities including potent activity against both chloroquine-sensitive (strain D6) and chloroquine-resistant *Plasmodium falciparum* (strain K1, W2) in vitro [10].

Despite extensive studies on the biological effects of cryptolepine, very little is known about its mechanisms of biotransformation in human and the widely used preclinical species, rat. The only work on the biotransformation of cryptolepine remains that of Stell et al. (2012) [11], who found cryptolepine to be oxidized by rabbit liver aldehyde oxidase forming cryptolepine-11-one which is inactive against *P. falciparum* in vitro. This finding could potentially limit the effectiveness of cryptolepine as an antimalarial agent, as aldehyde oxidase metabolism is often very rapid in human [12].

In previous pharmacokinetic reports by two independent researchers in rats and mice, cryptolepine showed a rapid disappearance from the plasma and localization in various tissues except the central nervous system and concluded that the hepatobiliary tract could be the main clearance pathway of cryptolepine [13, 14]. In contrast, McCurrie and colleagues [15] detected cryptolepine hydrochloride in the serum up to 10 h after oral administration of 10 mg/kg to rats and reported that no cryptolepine metabolites were detected in the serum samples. Similarly, Kuntworbe et al. (2013) [16] detected cryptolepine up to 24 h after 10 mg/kg intravenous administration to rats.

The lack of metabolic pathway information and the unclarity with regards to plasma metabolites and pharmacokinetics of cryptolepine led to the present study. This study aims at evaluating the metabolic profile and the mechanisms of biotransformation of cryptolepine by (1) characterization of metabolites following in vitro metabolism of cryptolepine in human and rat hepatocytes, (2) identification of the metabolites present in rat plasma and urine after single dose administration as well as (3) evaluation of the single-dose pharmacokinetics (PK) of cryptolepine in male Sprague Dawley (SD) rats.

## Materials and methods

### Reagents

Cryopreserved hepatocytes from human and rat were obtained from Celsis In Vitro Technologies (Baltimore, MD). Pooled plasma (K_2_ EDTA) from male Sprague Dawley rat was purchased from BioreclamationIVT (Westbury, NY). Cryptolepine hydrate and glyburide were purchased from Sigma Aldrich (St. Louis, MO). Acetonitrile, formic acid, and dimethyl sulfoxide were purchased from Thermo Fisher Scientific Inc. (Rockford, IL).

### In vitro metabolite profiling in hepatocytes

Mixed gender pooled cryopreserved, suspension hepatocytes were obtained from Bioreclamation IVT (catalogue numbers M00005 for rat and X008001 for human) and stored in the gas phase over liquid nitrogen until use. All experiments were carried out using Williams medium E supplemented with fetal bovine serum (10%). Stock solutions of test compounds (2 mM) were prepared in DMSO. Thawed hepatocytes (37 °C, 2 min) was transferred to a tube containing incubation medium (40 mL, 37 °C). The suspension was centrifuged for 1 min at 50 g at room temperature, and then the supernatant was removed and discarded. The hepatocyte pellet was resuspended in William medium E by gentle agitation in a small volume (2–5 mL) of incubation medium. An aliquot of cell suspension (50 μL) was mixed with trypan blue (50 μL) for viability assessment and cell counting. An appropriate volume of incubation medium was then added to the remaining cell suspension to give a final concentration 1 × 10^6^ viable cells/mL. Cell suspension (1 mL) was transferred to wells of a 12 well plate, and cryptolepine (10 μM) was added. The concentration of DMSO in the final wells were less than 0.1%. The samples were then incubated at 37 °C under an atmosphere of 75% O_2_, 5% CO_2_, 20% N_2_; 98% humidity with shaking (50 rpm) in a HERAcell 240i incubator (Thermo Fischer Scientific, Waltham, MA, USA). Incubations were initiated by the addition of cryptolepine to the hepatocytes. At each time point (0, 4, 24 h), 200 μL of incubation sample was added to 1 volume of chilled acetonitrile (0 °C), internal standard was added (8 μL, final concentration 5 μM), and the mixture was frozen at −80 °C.

### Analytical methods for metabolite profiling in hepatocytes

Samples were centrifuged at 10000 g at 4 °C for 5 min and supernatants (100 μL) were diluted with water (400 μL) and filtered (0.45 μm). Samples were analyzed by Capillary HPLC-MS/MS using a Chorus 200 binary syringe pump (CS Analytics, Beckenried, Switzerland), Triart C18 column (1.9 μm particle size, 150 mm × 0.3 mm) at 40 °C coupled to an LTQ Orbitrap XL mass spectrometer (Thermo Fisher Scientific, Waltham, MA, USA). Electrospray ionization in positive mode was used, recording full scans (m/z 150–1500) at resolution 30,000, and targeted or data dependent MS^n^ at unit resolution or high (30000) resolution as required for metabolite characterization. Chromatographic separation was achieved with the following mobile phases, (A): 10 mM ammonium formate in MS-grade water, MS-grade water/ acetonitrile (95:5), and 0.02% Trifluoroacetic acid; (B): MS-grade water/ methanol (5:95), 10 mM ammonium formate in MS-grade water, 0.02% Trifluoroacetic acid. A linear gradient of mobile phase B from 2 to 95% was applied over 25 min on the column at a flow rate of 4.5 μL/min. Experiments for the determination of exchangeable protons were performed by exchange of H_2_O by D_2_O (deuterated water) and CH_3_OH by CH_3_OD (deuterated methanol).

### Studies of cryptolepine in rats

Male Sprague Dawley rats, 320–340 g, purchased from Envigo Rms, Inc., Dublin, VA were used in the study. The experimental protocol was approved by the Novartis Institutes for Biomedical Research Cambridge Institutional Animal Care and Use Committee (approval August 2014). All rats were housed under constant environmental conditions (21 ± 2 °C, 40 ± 5% humidity, and 12-h light-dark cycles) and were allowed free access to food and water. The rats were fasted overnight (20 h) before oral dosing but food was returned to animals 4 h post *p.o.* dose.

Cryptolepine was administered intravenously and orally at doses of 1 and 5 mg/kg, respectively (*n =* 2 for each route of administration). Cryptolepine was dissolved in 1 N NaOH/PEG300/Cremophor EL/Solutol/phosphate buffered saline (1:30:5:5:59% volume) for intravenous administration (via a catheter in the left jugular vein). For oral administration, cryptolepine was suspended in Tween 80/methylcellulose/water (0.5:0.5:99.5% volume/weight/volume). Blood samples were collected from the animals at 5, 15, 30 min and 1, 2, 4, 7 and 24 h post intravenous dose and at 15 and 30 min and 1, 2, 4, 7 and 24 h post oral dose via a catheter in the right jugular vein. Rats were euthanized by cardiac puncture at the last blood collection time point. Urine was also collected at 0–7 and 7–24 h time intervals after both routes of administration. Plasma was obtained from blood samples by centrifugation at 8161 g for 2 min.

### In vivo metabolite profiling in SD rat urine and plasma

The plasma samples were pooled according to the Hamilton pooling method [17]. Pooled samples were extracted with 2 volumes of chilled acetonitrile with 0.1% formic acid. Thereafter, the supernatants volume was reduced to ca. 30 μL under a gentle stream of nitrogen gas. 2 mL of pooled urine samples (approximately 20% volume of each time point for each animal) were centrifuged at 4000 rpm for 3 min to remove any particles.

### Analytical methods for metabolite profiling in SD rat urine and plasma

Sample analysis and metabolite identification were carried out on a Thermo LTQ-Orbitrap mass spectrometer (Thermo Fisher Scientific, Waltham, MA) interfaced with a 3 Ti high-performance LC pump and CTC PAL autosampler (LEAP Technologies, Carrboro, NC). The analytes were separated on a Waters Symmetry C18 analytical column (5 μm particle size, 2.1 × 150 mm; Waters, Milford, MA) with a 35-min gradient elution method. The mobile phases consisted of (A) 10 mM ammonium formate in MS-grade water and (B) MS-grade acetonitrile. The sample aliquots were eluted at a flow rate of 0.25 mL/min with 10% B over 5 min. Mobile phase (B) was gradually increased to 90% over 24 min. The column was then returned to 10% B and held for 3 min before the next injection.

### Structural characterization of metabolites

The structural characterization of metabolites in plasma, urine and in vitro samples was carried out by MS/MS analysis after LC separation of analytes (described above).

High resolution MS and MS/MS spectra with collision-induced dissociation were obtained in positive ion mode. The structures of the metabolites were determined based on their elemental composition by exact mass measurement, MS/MS fragmentations, and number of exchangeable hydrogens based on hydrogen-deuterium exchange in deuterated solvent.

### Quantification of Cryptolepine in rat plasma and urine

Urine samples were diluted in 1 volume of blank rat plasma. All plasma and urine samples were then diluted 6-fold in acetonitrile containing internal standard (glyburide) and centrifuged (5000 g at 4 °C for 30 min) to precipitate proteins. The supernatant samples were then analysed by LC-MS/MS. The analytical system consisted of an API 4000 instrument mass spectrometer (AB Sciex, Foster City, CA), coupled to an Agilent 1200 system (Agilent Technologies, Inc., Santa Clara, CA) and a CTC HTS PAL autosampler (LEAP Technologies, Carrboro, NC). Analytes in plasma and urine samples were separated using an ACE C18 HPLC column (3 μm, 30 mm × 2.1 mm i.d. MAC-MOD Analytical, Inc. Chadds Ford, PA). The column was eluted using an isocratic gradient over 3.5 min with mobile phase consisting of 0.1% formic acid in water (solvent A) and 0.1% formic acid in acetonitrile (solvent B) at a flow rate of 0.7 mL/min. Cryptolepine was detected by multiple reaction monitoring (MRM) transition of 233 → 190 under positive ion mode. The internal standard, glyburide, an MRM transition of 494 → 169 was used. Linearity of calibration curves of cryptolepine were confirmed between 0.1 and 5000 ng/ml.

### Pharmacokinetic analysis

Non compartmental pharmacokinetic analysis was performed using Phoenix 6.3 (Certara, St Louis, MO, USA) to determine the following pharmacokinetic parameters; area under the plasma concentration time curve (AUC), maximum plasma concentration (C_max_), time to reach C_max_ (T_max_), plasma clearance (CL_p_), steady state volume of distribution (V_ss_), elimination half-life of plasma concentration (t_1/2_), mean residence time of plasma concentration (MRT) and oral bioavailability (% F). Amount excreted in urine (Ae) was calculated from measured urine concentrations and volumes.

## Results

### Hepatocyte incubation and metabolite identification

**Fig. 2:**
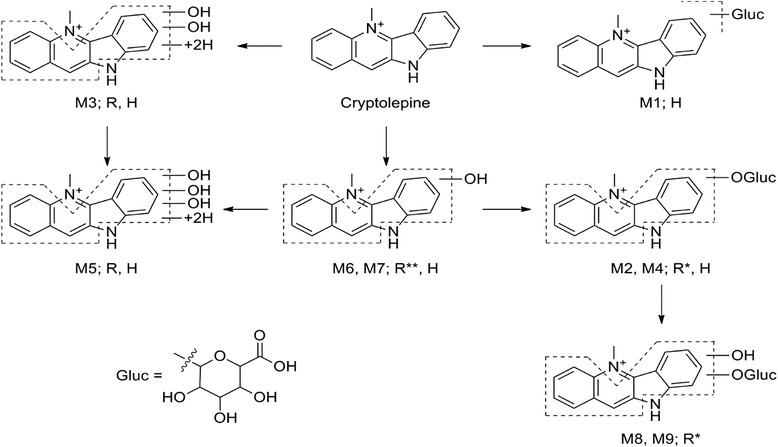
Proposed metabolite structures and metabolic pathways for cryptolepine determined by LC-MS/MS after incubation with rat and human hepatocytes for up to 24 h and in plasma and urine of SD rats after single dose oral administration of cryptolepine. R: detected after rat hepatocyte incubation; H: detected after human hepatocyte incubation. * Metabolite detected in rat urine; ** metabolite detected in rat urine and plasma

**Fig. 3:**
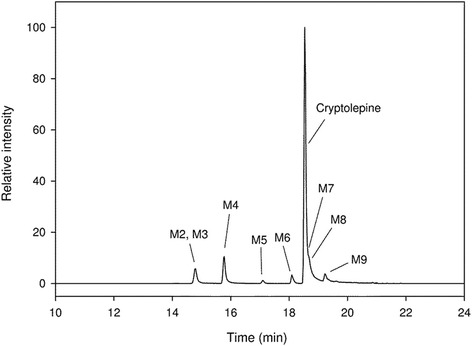
Representative extracted ion chromatogram for cryptolepine and proposed metabolites formed in rat hepatocytes after 24 h of incubation. M1 is not shown as it was only detected in the human hepatocyte incubation

**Fig. 4:**
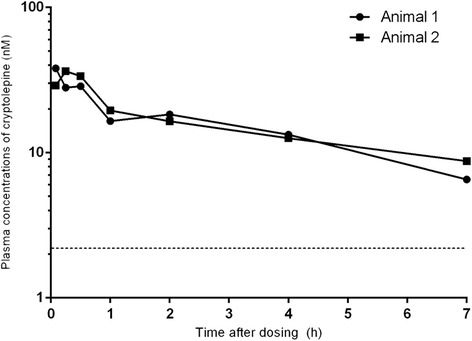
Plasma concentration-time profiles of cryptolepine after single intravenous (1 mg/kg) administrations to Sprague Dawley rats. The lower dotted line indicates the LLOQ (2.2 nM) of LC–MS/MS method for quantifying cryptolepine in rat plasma

Metabolites were identified in hepatocyte incubates by analysis of high resolution LC-MS/MS data. Nine metabolites were identified in human and rat hepatocytes (see Table 1 for MS data and Figs. 2 and 3 for proposed structures and a representative mass chromatogram) resulting from metabolic pathways mainly involving hydroxylation (M2, M4, M6, M7, M8, M9), proposed dihydrodiol formation (M3, M5) and glucuronidation (M1, M2, M4, M8, M9). All metabolites were detected in hepatocyte incubations from both species except for glucuronide M1, which was only formed by human hepatocytes, and metabolites M8 and M9, which were only formed by rat hepatocytes. Based on the proposed metabolism pathways (Fig. 4), the apparent primary metabolites are hydroxylation (M6/M7), dihydrodiol formation (M3), and glucuronidation (M1).

**Table 1:** LC-MS data for cryptolepine and proposed metabolites

Metabolite code	Retention time (min)	Measured m/z	Proposed MH+ formula	Product ions m/z and proposed neutral loss formula	m/z after H/D exchange	Detected in hepatocyte species	Detected in rat plasma/urine
M1	12.9	409.1394	C_22_H_21_N_2_O_6_	233 (-C_6_H_9_O_6_); MS3 218 (-CH_3_)	413	H	
M2	14.8	425.1344	C_22_H_21_N_2_O_7_	249 (-C_6_H_9_O_6_); MS3 221 (-CO), 234 (-CH_3_)	430	R, H	Urine
M3	14.8	267.1129	C_16_H_15_N_2_O_2_	221 (-CH_2_O_2_), 239 (-CO), 249 (-H_2_O), 252 (-CH_3_)	270	R, H	
M4	15.8	425.1343	C_22_H_21_N_2_O_7_	249 (-C_6_H_9_O_6_); MS3 221 (-CO), 234 (-CH_3_)	430	R, H	
M5	17.1	283.1078	C_16_H_15_N_2_O_3_	211 (-C_3_H_4_O_2_), 225 (-C_3_H_6_O), 237 (-CH_2_O_2_), 239 (-C_2_H_4_O), 255 (-CO), 265 (-H_2_O), 268 (-CH_3_)	287	R, H	
M6	18.1	249.1023	C_16_H_1__3_N_2_O	221 (-CO), 231 (-H_2_O), 234 (-CH_3_), 235 (-CH_2_)	251	R, H	Urine, plasma
Cryptolepine	18.5	233.1073	C_16_H_13_N_2_	218 (-CH_3_), 219 (-CH_2_)	234	R, H	Urine
M7	18.6	249.1022	C_16_H_13_N_2_O	221 (-CO), 231 (-H_2_O), 234 (-CH_3_), 235 (-CH_2_)	251	R, H	
M8	18.7	441.1294	C_22_H_21_N_2_O_8_	222 (-C_6_H_9_O_6_ - C_2_H_3_O), 237(-C_6_H_9_O_6_ - CO), 247(-C_6_H_9_O_6_ - H_2_O), 250(-C_6_H_9_O_6_ - CH_3_), 265 (-C_6_H_9_O_6_)	447	R	
M9	19.2	441.1292	C_22_H_21_N_2_O_8_	222 (-C_6_H_9_O_6_ - C_2_H_3_O), 237(-C_6_H_9_O_6_ - CO), 247(-C_6_H_9_O_6_ - H_2_O), 250(-C_6_H_9_O_6_ - CH_3_), 265 (-C_6_H_9_O_6_)	447	R	Urine

R: rat; H: human

### Metabolites in rat urine and plasma

Plasma and urine following oral administration of 5 mg/kg cryptolepine were also analyzed for metabolites by LC-MS/MS. All of the metabolites identified were also detected in rat hepatocyte incubations. A total of three metabolites were detected in SD rat urine (M2, M6, M9) and one metabolite was detected in plasma (M6). Metabolism included; oxidation (M6), oxidation and glucuronidation (M2), and di-hydroxylation followed by glucuronidation (M9). Metabolites M2 and M9 were observed in urine only.

### Pharmacokinetic profile of cryptolepine in rat

**Fig. 5:**
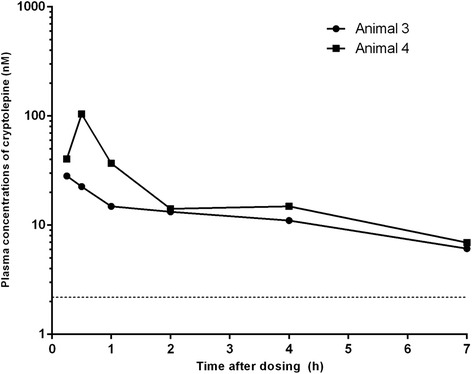
Plasma concentration-time profiles of cryptolepine after single oral (5 mg/kg) administration to Sprague Dawley rats. The lower dotted line indicates the LLOQ (2.2 nM) of the LC–MS/MS method for quantifying cryptolepine in rat plasma

The pharmacokinetics of cryptolepine in rat were investigated following 1 mg/kg intravenous and 5 mg/kg oral administration. The plasma concentration-time profiles for cryptolepine are shown in Figs. 4 and 5, and pharmacokinetic parameters are shown in Tables 2 and 3. Following both intravenous and oral administration, plasma concentrations were quantifiable up to and including the 7 h sampling time point, and pharmacokinetics were calculated based on a Tlast of 7 h. Plasma exposure was low and the average plasma clearance (CL_p)_ was high. Cryptolepine was extensively distributed as indicated by the high volume of distribution (Vss), resulting in a moderate plasma t_1/2_. Absorption after oral administration was fast with Cmax values of 28 nM and 104 nM reached within 0.25 and 0.5 h in animals 3 and 4 respectively. Oral bioavailability was low (16% and 28% in animals 3 and 4, respectively). Less than 1% of dose was excreted unchanged in urine within 24 h post dosing.

**Table 2:** Pharmacokinetic parameters of cryptolepine in rats following intravenous administration at a dose of 1 mg/kg

Animal ID	Plasma AUC_0–7h_ (nM.h)	CL_p_ (mL/min/kg)	Vss (L/Kg)	t_1/2_ (h)	MRT (h)	Urine Ae_0-24h_ (% dose)
1	106	521	147	3	5	0.22
2	109	402	180	6	7	0.84

AUC_0–7h_, the area under the concentration-time curve up to the last measurable time point in plasma; CL_p,_ plasma clearance; V_ss_, steady-state volume of distribution; t_1/2_, elimination half-life; T_max_; MRT, mean residence time; Ae_0-24h_, amount excreted within 24 h following administration

**Table 3:** Pharmacokinetic parameters of cryptolepine in rats following oral administration at a dose of 5 mg/kg

Animal ID	Plasma AUC_0–7_ (nM.h)	Cmax (nM)	Tmax (h)	F %	Urine Ae_0-24h_ (% dose)
3	83	28	0.3	16	0.15
4	146	104	0.5	28	0.12

AUC_0–7h_, the area under the concentration-time curve up to the last measurable time point in plasma; Cmax, maximum plasma concentration; Tmax, time to reach Cmax; F, oral bioavailability; Ae_0-24h_, amount excreted within 24 h following administration

## Discussion

The results of our present investigation with cryptolepine shed light on the metabolic profile and the mechanisms of biotransformation of cryptolepine in vitro in human and rat hepatocytes and in vivo in Sprague Dawley rats. Commercially available metabolically active hepatocytes have been shown to contain the complete complement of drug-metabolizing enzymes and hence serves as the closest in vitro surrogate for in vivo hepatic metabolism [18]. The metabolic profiles in rat and human were compared to assess potential metabolic clearance pathways, and whether all metabolites observed in human matrices were detected in the rodent species. Incubation of cryptolepine in rat and human hepatocytes for 24 h showed nine major metabolites. These identified metabolites lead to the proposed metabolism pathway shown in Fig. 2, with primary metabolism pathways in human and rat being hydroxylation and dihydrodiol formation, as well as glucuronidation in human. In rat plasma, only hydroxylation metabolite M6 was detected, and in rat urine M6 as well as secondary glucuronide metabolites of M6 (M2 and M9) were detected. This data suggests that M6 is a relevant in vivo metabolism pathway, at least in rat. In the absence of bile or feces metabolism data, further interpretations such as the relevance of metabolic clearance via dihydrodiol M3 cannot be made.

All human in vitro metabolites were also found in rat hepatocytes, except for metabolite M1. This metabolite is an N-glucuronide and is found in human, but not rat hepatocytes, indicating the possible involvement of a human specific UDP-glucuronosyltransferase (UGT) [19] in the metabolism of cryptolepine. According to the MIST and ICH M3 (R2) health authority guidances [20], major metabolites in human should be present in toxicology species also, with the exception of most phase II metabolites. As such, glucuronide M1 would be excluded from this assessment. With the similarities in the metabolites in human and rat, the rat model therefore offers an appropriate model for pre-clinical studies of cryptolepine.

In previous studies by Stell et al. (2012) [11], the involvement of aldehyde oxidase in the metabolism of cryptolepine was proposed, leading to the formation of metabolite cryptolepine-11-one. The metabolites M6/M7 identified in this study in both human and rat hepatocytes are formed by oxidative hydroxylation indicating that involvement of aldehyde oxidase is possible. Metabolite (M6) was also detected in rat plasma samples after oral administration of cryptolepine. Aldehyde oxidase metabolism has in the past caused difficulties in drug development, as it is often more active in human than in preclinical species. For example, a p38 kinase inhibitor (RO1) for the treatment of rheumatoid arthritis was terminated because of unexpected rapid clearance and short half-life in man, proposed to be due to aldehyde oxidase metabolism [12]. The aldehyde oxidase and N-glucuronidation pathways could potentially result in significantly different clearance and/or disposition in human compared to rat, depending on the extent of formation and stability of each metabolite in humans in vivo. The dihydroxylation metabolites (M3/M5) are likely to be formed via initial epoxide formation, indicating the possibility of cytochrome P450 metabolism of cryptolepine**.** The proposed dihydrodiol metabolites M3 and M5 were not found in rat in vivo, but may be formed in the liver and excreted in feces.

Following intravenous and oral administration of cryptolepine, rats exhibited high plasma clearance, extensive distribution and low oral bioavailability. Elimination of the unchanged drug in urine was negligible, suggesting that renal elimination of unchanged drug is not a relevant pathway of elimination. This is not surprising given the physicochemical properties of cryptolepine, a relatively lipophilic molecule (cLogP 4.3) which may lead to a predisposition for elimination by metabolism [21, 22].

The in vivo study design used a single low dose of cryptolepine to 2 animals per administration route. Due to the relatively low concentrations in plasma, cryptolepine was only quantifiable up to 7 h post-dose. It is possible that due to this limitation, the full PK profile of cryptolepine may not have been captured in vivo. However, the present investigation provides an additional assessment of rat PK, in the same animals used for metabolite identification. This in vivo study however was a mere proof of concept hence the low sample size used. More in depth radiolabelled cryptolepine and higher dose studies allowing analysis of plasma and excreta over a longer timeframe similar to mass balance excretion studies as described in literature [23] may add further in-depth knowledge of the absorption, distribution, metabolism and excretion characteristics of cryptolepine in rats.

Kuntworbe et al. (2013) [16] also showed an extensive distribution of cryptolepine, with observed distribution into the spleen, heart, lungs, kidney and liver coupled with slow clearance from these tissues. Based on this data and our new rat pharmacokinetic data, cryptolepine is likely to extensively distribute in human, possibly accumulating in vital organs and extending plasma residence time. This property would be particularly advantageous for the clearance of erythrocytic stage parasites, as accumulation of antimalarial compounds into the food vacuole of the plasmodium parasite has been associated with the efficacy of these compounds [24].

## Conclusions

In conclusion, this study revealed substantial information about the metabolism of cryptolepine in rat and human, as well as in vivo in SD rat. In rat and human hepatocytes, nine metabolites were observed, with hydroxylation, dihydrodiol formation and glucuronidation proposed to be the major metabolic pathways. Metabolites were qualitatively similar between rat and human, and the metabolites found in the rat urine and plasma also found in vitro, indicating that rat is likely to be an appropriate preclinical species for cryptolepine studies. Rat PK data indicated that cryptolepine was cleared quickly from plasma, was extensively distributed and was not extensively eliminated unchanged in the urine. The data suggest that metabolism is likely a pathway of elimination of cryptolepine but that renal elimination is negligible.

## Abbreviations

%F: oral bioavailability
Ae: Amount excreted in urine
AUC: Area under plasma concentration versus time curve
CL_p_: plasma clearance
DMSO: dimethyl sulfoxide
*i.v.*: intravenous administration
LC: Liquid chromatography
MRM: Multiple reaction monitoring
MRT: Mean residence time of plasma concentration
MS: Mass spectrometry
MS/MS: Tandem mass spectrometry
*p.o*.: oral administration
PK: pharmacokinetics
*t*_1/2_: Elimination half-life of plasma concentration
T_max_: Time to reach the maximum plasma concentration (C_max_)
UGT: UDP-glucuronosyltransferase
V_ss_: steady state volume of distribution
WHO: World Health Organization
